# Altered Functional and Causal Connectivity of Cerebello-Cortical Circuits between Multiple System Atrophy (Parkinsonian Type) and Parkinson’s Disease

**DOI:** 10.3389/fnagi.2017.00266

**Published:** 2017-08-10

**Authors:** Qun Yao, Donglin Zhu, Feng Li, Chaoyong Xiao, Xingjian Lin, Qingling Huang, Jingping Shi

**Affiliations:** ^1^Department of Neurology, Affiliated Brain Hospital of Nanjing Medical University Nanjing, China; ^2^Department of Radiology, Affiliated Brain Hospital of Nanjing Medical University Nanjing, China

**Keywords:** functional connectivity, granger causality analysis, multiple system atrophy, Parkinson’s disease, resting-state fMRI

## Abstract

Lesions of the cerebellum lead to motor and non-motor deficits by influencing cerebral cortex activity via cerebello-cortical circuits. It remains unknown whether the cerebello-cortical “disconnection” underlies motor and non-motor impairments both in the parkinsonian variant of multiple system atrophy (MSA-P) and Parkinson’s disease (PD). In this study, we investigated both the functional and effective connectivity of the cerebello-cortical circuits from resting-state functional magnetic resonance imaging (rs-fMRI) data of three groups (26 MSA-P patients, 31 PD patients, and 30 controls). Correlation analysis was performed between the causal connectivity and clinical scores. PD patients showed a weakened cerebellar dentate nucleus (DN) functional coupling in the posterior cingulate cortex (PCC) and inferior parietal lobe compared with MSA-P or controls. MSA-P patients exhibited significantly enhanced effective connectivity from the DN to PCC compared with PD patients or controls, as well as declined causal connectivity from the left precentral gyrus to right DN compared with the controls, and this value is significantly correlated with the motor symptom scores. Our findings demonstrated a crucial role for the cerebello-cortical networks in both MSA-P and PD patients in addition to striatal-thalamo-cortical (STC) networks and indicated that different patterns of cerebello-cortical loop degeneration are involved in the development of the diseases.

## Introduction

The parkinsonian variant of multiple system atrophy (MSA-P) is a neurodegenerative disorder that is clinically difficult to differentiate from idiopathic Parkinson’s disease (PD), especially in the early stages of the diseases (Wenning et al., 2000; Kim et al., 2016). Inchoate differentiation between MSA-P and PD has significant therapeutic and rehabilitative implications. At the neuronal level, these diseases are all characterized by extensive cell loss in the substantia nigra pars compacta (Dickson, 2012). In the past, functional brain imaging had been proved to be of some value for the differential diagnosis of parkinsonism. Positron emission tomography, for instance, disclosed decreased striatal presynaptic uptake, binding, glucose metabolism, and post-synaptic binding in both MSA-P and PD (Ghaemi et al., 2002; Bohnen et al., 2006), especially the reduced post-synaptic binding in MSA-P. Liu et al. (2013) described that the dopamine deficits in striatal subregions impair the function of striatal-thalamo-cortical (STC) networks involved in motor, cognitive and emotional processing (Braak and Braak, 2000).

Considering the demonstrated alteration of pathology was found in the basal ganglia of PD and MSA-P (Galvan et al., 2015). The cerebellum is also an important component in motor control, higher cognitive, and emotional processing (Allen et al., 2005; Schutter and van Honk, 2005; Habas, 2010). It is known to affect cerebral cortical activity by cerebello-thalamo-cortical (CTC) circuits (Middleton and Strick, 2000). Previous studies have demonstrated the cerebellum to be involved in these diseases. For example, in MSA-P patients, morphological and microstructural alterations of cerebellum have been reported (Nicoletti et al., 2006a,b). The cerebellar functional activation was increased in MSA-P after repetitive transcranial magnetic stimulation (rTMS) treatment (Wang H. et al., 2016). Moreover, the cerebellum is anatomically and functionally connected with the basal ganglia and its connectivity changes in PD have been discovered (Wu and Hallett, 2013). Wu et al. (2009) found increased cerebellar activity in PD by a regional homogeneity method. Another causal connectivity study has shown that the connectivity of cortico-cerebellar motor regions is strengthened in PD during the performance of self-initiated movement (Wu et al., 2011). A PET research has also revealed that the cerebellum is a crucial node in the abnormal metabolic patterns of both MSA-P and PD (Poston et al., 2012), with decreased cerebellar 18F-fluorodeoxyglucose metabolism in MSA-P and increased in PD. It has been presumed that compensatory activity in CTC circuits in PD patients may act as a compensatory mechanism to overcome the deficits in the STC circuits (Cerasa et al., 2006; Palmer et al., 2010). However, the exact role of the cerebellum in Parkinsonism, especially the MSA-P, remains unclear.

The cerebellar outputs polymerize to the dentate nucleus (DN), which successively sends neural fibers to the thalamus and cerebral cortex via the superior cerebellar peduncles, thus completing the cerebello-cortical circuits (Middleton and Strick, 2000). Histological studies have demonstrated anatomically segregated cerebello-cortical circuits including motor and non-motor loops (Clower et al., 2001; Middleton and Strick, 2001). Available evidence regarding the cerebello-cortical circuits connecting the lateral cerebellum to motor and non-motor cortical areas is limited in humans, due to technical challenges in assessing the long polysynaptic connections between the cerebellum and the cerebral cortex *in vivo*. Although the established cerebellar involvement in Parkinsonism, subtile studies on cerebellum for the differential diagnosis of parkinsonian syndromes are still penurious to date. Recently, resting-state fMRI (rs-fMRI) has been widely used to discover abnormalities in spontaneous neuronal activity by measuring the functional connectivity between spatially distinct brain regions (Biswal et al., 1995). Functional connectivity (FC) is defined as statistical dependencies among remote neurophysiological events. However, granger causality analysis (GCA) is another widely used method for identifying directed functional (‘causal’) connectivity in neural time series data (Roebroeck et al., 2005). GCA has been applied to human EEG data (Hesse et al., 2003). Moreover, GCA has recently also been applied to human fMRI data based on temporal order (Friston, 2009; Seth et al., 2013). It has been widely used in exploring cognitive functions such as working memory (Protopapa et al., 2014, 2016), as well as other neurological disorders (Brovelli et al., 2004; Jiao et al., 2011). The DN is the largest single structure linking the cerebellum to the rest of the brain. Accordingly, we selected the bilateral DN as regions of interest (ROIs) to explore the different roles of the cerebellum in PD and MSA-P.

Only a few studies have investigated DN connectivity changes in the resting state in PD (Liu et al., 2013; Ma et al., 2015). However, there has been no reported data about MSA-P. In this study, we focused on potential connectivity changes between the DN and cerebral cortices. We hypothesized that the connectivity between the DN and cortical or subcortical regions may be altered and implicate motor symptoms difference between PD and MSA-P. To test this hypothesis, the functional connectivity (FC) and multivariate granger causality analysis (mGCA) methods were combined to explore the connectivity differences within the cerebello-cortical circuits during resting state.

## Materials and Methods

### Participants

All subjects were recruited from Nanjing Brain Hospital from June 2013 to December 2015. Twenty-six MSA-P patients, 31 PD patients and 30 normal subjects were recruited into this study. The patients were diagnosed by a movement disorders specialist using established criteria: PD based on United Kingdom PD Society Brain Bank criteria (Hughes et al., 1992) and probable MSA-P based on the American Academy of Neurology and American Autonomic Society criteria (Gilman et al., 2008). Five subjects (2 MSA-P, 2 PD, and 1 control) were excluded due to excessive head motion during the fMRI procedure or incomplete scanning data, yielding a total of 24 MSA-P patients, 29 PD patients and 29 controls for the final analysis. All subjects underwent comprehensive neuropsychological assessments. Overall cognitive condition was assessed by the Mini-Mental State Examination (MMSE), Montreal Cognitive Assessment (MoCA) and Frontal Assessment Battery (FAB). The severity of motor symptoms for all patients was assessed using the motor part of Unified Parkinson’s Disease Rating Scale (UPDRS-III) and the Hoehn-Yahr (H-Y) scale. In this study, we used the sum of all left hemibody Part III items 20–26 (UPDRS-III L), the sum of all right hemibody Part III items 20–26 (UPDRS-III R) and the sum of all Part III items (UPDRS-III total). In addition, MSA-P patients were evaluated by the Unified Multiple System Atrophy Rating Scale (UMSARS), which was conducive to classification. Assessments were performed on the day before fMRI scanning in all subjects. Patients who had hemorrhage, infarction, tumors, trauma, or severe white matter hyperintensity were excluded from the study. All participants had written informed consent and the study was approved by the Medical Research Ethical Committee of Nanjing Brain Hospital, Nanjing, China.

### Image Acquisition

All scans were acquired using a Siemens 3.0 T singer scanner (Siemens, Verio, Germany) with an 8-channel radio frequency coil. All subjects lay supine with their head cozily fixed by sponge earplugs to minimize head movement. Participants were instructed to remain as still as possible, close their eyes, remain awake and not think of anything. Three-dimensional T1 weighted images were acquired in a sagittal orientation employing a 3D-SPGR sequence with the following parameters: TE = 3.34 ms; TR = 2530 ms; flip angle = 7°; 128 sagittal slices; 1.33 mm slice thickness; matrix = 256 × 256. Functional images were collected using a gradient-recalled echo-planar imaging pulse sequence: 140 time points (that were sufficient to assess resting state connectivity); TE = 30 ms; TR = 2000 ms; FOV = 240 mm × 240 mm; matrix = 64 × 64; flip angle = 90°; 30 axial slices; 3.0 mm thickness; section gap = 0 mm.

### Definition of Regions of Interest (ROIs)

Regions of interest of the left and right DN were defined by WFU PickAtlas^1^ and were resliced into Montreal Neurological Institute (MNI) space. The blood oxygen level dependent (BOLD) time series of the voxels within the ROI were extracted to generate the reference time series for each ROI.

### Data Preprocessing

The fMRI data were preprocessed using Data Processing Assistant for Resting-State fMRI toolkit (DPARSF^2^), which is based on the Statistical Parametric Mapping software SPM8^3^. The first 10 volumes of the rest session were discarded for each subject. The remaining images were corrected for slice timing and motion correction. According to the record of head motion, all participants had less than 2.0 mm maximum displacement in the x, y, or z plane and less than 2° of angular rotation about each axis. After spatial normalization to T1 space, all images were resampled into 3 mm × 3 mm × 3 mm voxels and spatially smoothed with a Gaussian filter of 4 mm full-width at half-maximum (FWHM). The fMRI data were then temporally band-pass-filtered (0.01–0.08 Hz) to remove low-frequency drifts and physiological high-frequency noise. To further reduce the effects of confounding factors, linear drift, six motion parameters and the mean time series of all voxels within the entire brain, white matter and cerebrospinal fluid signals were removed from the data by linear regression.

In the fMRI data, global signal can be defined as the time series of signal intensity averaged across all brain voxels, which includes both the signal of the neural activity and the noise of the non-neural activity. Global signal regression (GSR) uses linear regression to remove variance between the global signal and the time course of each individual voxel. It can improve the specificity of positive correlations and improve the correspondence to anatomical connectivity. It helps remove non-neuronal sources of global variance such as respiration and movement. With the growing use of GSR, some problems it brings have led to some controversy. Different processing techniques likely produce different complementary insights into the brain’s functional organization. Whether GSR should be useful or not depend on the scientific question and how we use it. If applied and interpreted correctly, they provide complementary information (Murphy and Fox, 2016). In this study, we are concerned with the neural activity of a particular brain area, and are not interested in the noise of those non-neural activities, so we applied GSR to remove global signal variance from all voxel time series.

The voxel based morphometry (VBM) was processed and examined using SPM8 software. The 3D-T1 weighted images were segmented into gray matter, white matter and cerebrospinal fluid. The gray matter and white matter images were then normalized and resampled to MNI space in 1.5 mm cubic resolution with modulation to preserve the local tissue volumes. The resulting images were smoothed using an 8 mm FWHM Gaussian kernel.

### Statistical Analysis

To compare certain demographic information and clinical characteristics (age, education, MMSE, MoCA, FAB), one-way analysis of variance (ANOVA) was used. Disease duration, UPDRS-III, H-Y, and levodopa equivalent daily dose (LEDD) were compared using the two-sample *t-*test. A chi-squared test was used to compare sex distribution among the groups. VBM analysis among MSA-P, PD, and control groups was carried out with the ANOVA, followed by Bonferroni test for *post hoc* comparisons. All data were statistically analyzed using SPSS19.0 (SPSS, Inc., Chicago, IL, United States). Two-sided values of *p* < 0.05 were considered statistically significant.

### Functional Connectivity Analysis

Functional connectivity analysis was performed between each seed reference and the entire brain in a voxel-wise manner using the REST Toolkit^2^. Correlation coefficients were transformed to z-values using the Fisher r-to-z transformation to enhance normality. Statistical analysis across the three groups was conducted using one-way analysis of covariance (ANCOVA), with age, gender, disease duration, and gray matter volume as covariates. Then, *post hoc* two-sample *t*-tests were followed. The multiple comparisons of ANCOVA result was AlphaSim corrected with a cluster-level significance threshold of *p* < 0.01 (cluster size > 32 voxels and voxel-level *p* < 0.01; determined by a Monte-Carlo simulation). The *post hoc* two-sample *t-*tests were performed within a mask showing conspicuous differences acquired from the ANCOVA results, with a significance threshold of *p* < 0.01 with AlphaSim correction (cluster size > 14 voxels; voxel-level *p* < 0.01; determined by a Monte-Carlo simulation). The ANOVA was also performed with the similar results of ANCOVA after correction.

### Granger Causality Analysis

Causal connectivity refers to the influence that one neural system exerts over another. The distinction between functional and causal connectivity is that functional connectivity is ambiguous with respect to underlying directed interactions that generated the observed correlations. While causal connectivity corresponds to the intuitive notion of coupling or directed causal influence (Friston, 2011). GCA is a feasible technique for analyzing fMRI data (Wen et al., 2013). It is based on the idea that, given two time series x and y, if knowing the past of y is useful for predicting the future of x, then y must have a causal influence on x. GC method shows distinct and complementary functions in relation to the detection of causality and does not need predefined model. To identify the informational influence on the functional interactions and infer their causal relationship within the cerebello-cortical circuits, the mGCA method was applied (Blinowska et al., 2004; Chen et al., 2014). This approach is based on the MATLAB toolbox (The MathWorks, Inc., Natick, MA, United States). Briefly, to determine the structure of the cerebello-cortical circuits, nine ROIs were selected according to the abnormal functional connectivity patterns identified in the group comparisons in this study (for details refer to **Table 1**). Each seed region was represented by a radius of 6 mm around the central coordinates. The average time series for each ROI was extracted and may be expressed in Eq. 1.

$$X(t)=\left(x_{1}(t),x_{2}(t),...,x_{m}(t)\right)$$

**Table 1:** Brain regions for granger causality analysis across all subjects.

ROIs	Brain regions	Sides (L/R)	MNI coordinates		
			
			*X*	*Y*	*Z*
ROI 1	Posterior cingulate cortex	–	-3	-36	33
ROI 2	Medial prefrontal cortex	–	3	42	39
ROI 3	Dorsal lateral prefrontal cortex	L	-33	49	9
ROI 4	Dorsal lateral prefrontal cortex	R	48	27	27
ROI 5	Precentral gyrus	L	-51	9	39
ROI 6	Inferior parietal cortex	L	-34	-62	46
ROI 7	Inferior parietal cortex	R	39	-66	45
ROI 8	Cerebellar dentate nucleus	L	-16	-54	-32
ROI 9	Cerebellar dentate nucleus	R	15	-56	-30


*Brain regions obtained from differential functional connectivity of cerebello-cortical network among MSA-P, PD, and controls; MNI, Montreal Neurological Institute; L, left; R, right.*

where m represented the number of ROIs. The values of causal connectivity strengths from all other regions to region j were measured by multivariate auto regression (MVAR) model (Eq. 2).

$$x_{j}(t)=\overset{p}{\underset{i=1}{\Sigma }}A_{j}(i)X(t-i)+E_{j}(t)$$

The parameter *p* is the model order or the lag parameter. *A_j_*(*i*) is the regression coefficient matrix, *X* is the time series matrix of different regions, *E* is the residual error matrix. The optimal lag parameter *p* is usually determined by minimizing Akaike Information Criterion (AIC) (Akaike, 1998). For each subject, random-effect Granger causality maps were calculated. Statistical thresholds for these maps was performed in the context of the bootstrap technique with corrections for multiple comparisons based on a permutation test (*p* < 0.05) (Chen et al., 2014). For each group, an average Granger causality map was created to illustrate the effective connectivity influence on the paired regions. Then, group comparisons were also conducted to identify the significantly altered causal influence between paired brain areas using the Kruskal–Wallis, followed by the Dunn–Bonferroni test for *post hoc* comparisons.

### Connectivity-Behavior Correlation

To explore whether the alterations of causal connectivity are covariant with disease progression, a correlation analysis between altered causal connectivity and neuropsychological performance metrics, disease duration and LEDD was performed separately for MSA-P and PD patients. First, causal connectivity values for the significant group differences were extracted. Then, Pearson’s correlative analysis was conducted to examine the relationships between causal connectivity and neuropsychological scores [including MMSE, MoCA, FAB, UPDRS-III (total, L, R) and H-Y staging scale] and LEDD.

## Results

### Demographic and Clinical Characteristics

The demographic and clinical characteristics of the participants are presented in **Table 2**. There were no significant differences in age, gender, education level, MMSE, and MoCA scores among the three groups. No significant differences were found in UPDRS-III (total, L, R) and H-Y staging scores between MSA-P and PD groups. Difference in disease duration was significant between MSA-P and PD (*p* < 0.001). The FAB scores were significantly different between MSA-P versus control (*p* = 0.001) and PD versus control (*p* = 0.037).

**Table 2:** Demographic and clinical characteristics of the participants.

Groups	MSA-P	PD	Control	*p-*Value
	(*n* = 24)	(*n* = 29)	(*n* = 29)	
Age, years	63.00 ± 6.99	66.52 ± 6.98	63.21 ± 8.01	0.141
Gender, male/female	13/11	18/11	12/17	0.282
Education, years	8.45 ± 1.18	8.52 ± 1.09	8.41 ± 1.21	0.944
Disease duration years	2.37 ± 0.92	4.62 ± 1.26	NA	<0.001^a^
GMV, ml	489.01 ± 49.59	511.77 ± 54.47	521.38 ± 41.75	0.064
WMV, ml	515.92 ± 62.10	528.05 ± 53.29	541.06 ± 68.25	0.337
MMSE	27.71 ± 0.86	27.59 ± 1.40	27.93 ± 0.88	0.373
MoCA	25.17 ± 1.43	24.79 ± 1.40	25.58 ± 1.21	0.087
FAB	14.63 ± 1.10	15.00 ± 1.22	15.72 ± 0.88	0.001^bc^
UPDRS-III, total	28.42 ± 7.22	24.45 ± 9.01	NA	0.087
UPDRS-III, right	8.08 ± 5.05	7.45 ± 3.33	NA	0.586
UPDRS-III, left	7.58 ± 3.94	6.51 ± 2.63	NA	0.245
H-Y	2.79 ± 0.76	2.43 ± 0.80	NA	0.102
LEDD, mg/day	543.23 ± 100.8	504.31 ± 85.65	NA	0.135


*GMV, gray matter volume; WMV, white matter volume; MMSE, Mini-Mental State Examination; MoCA, Montreal Cognitive Assessment; FAB, Frontal Assessment Battery; UPDRS-III, Unified Parkinson’s Disease Rating Scale-motor part III; H-Y, Hoehn-Yahr staging scale; LEDD, levodopa equivalent daily dose; NA, not applicable; *p* < 0.05 was considered significant. ^a^Means significant difference between the two groups (MSA-P versus PD). ^bc^Means significant group differences between (MSA-P versus control), (PD versus control) by *post hoc* comparisons.*

### Voxel Based Morphometry

Voxel based morphometry did not reveal significant differences between patients and controls for gray matter volume and white matter volume (for details refer to **Table 2**).

### Functional Connectivity

**FIGURE 1:**
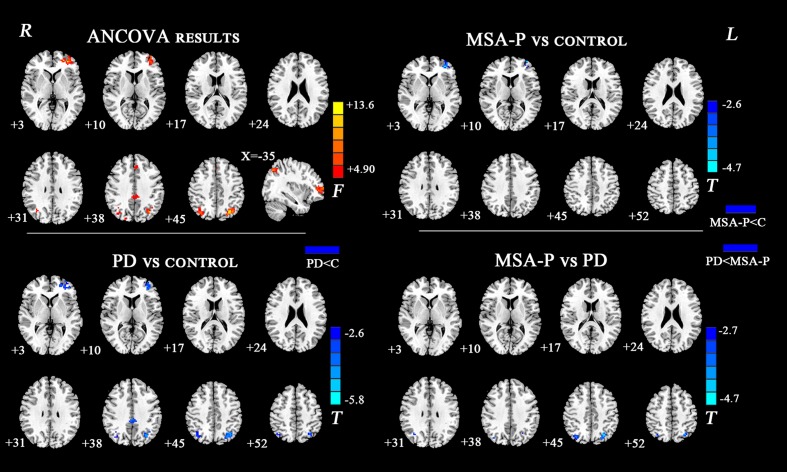
Statistical maps showing FC differences of left DN in different brain regions among the MSA-P, PD, and control groups. The threshold for display was set to *p* < 0.01 corrected by AlphaSim; Results are in MNI space; Red color represents the increased correlation while the blue color represents the decreased correlation; R, right; L, left; C, controls.

**FIGURE 2:**
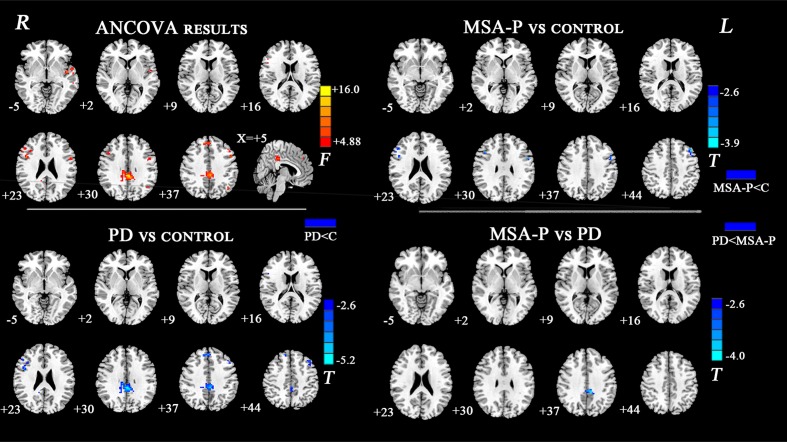
Statistical maps showing FC differences of right DN in different brain regions among the MSA-P, PD, and control groups. The threshold for display was set to *p* < 0.01 corrected by AlphaSim; Results are in MNI space; Red color represents the increased correlation while the blue color represents the decreased correlation; R, right; L, left; C, controls.

The ANCOVA results revealed that the left DN had significantly different FC values in the frontal, parietal or cingulate cortices among MSA-P, PD, and controls (**Table 2** and **Figure 1**). A further detailed investigation of these alterations in the three groups showed that the FC values of both MSA-P and PD patients were significantly decreased in the left dorsolateral prefrontal cortex (DLPFC) compared with the controls. PD patients exhibited lower FC values in the bilateral inferior parietal lobe compared to MSA-P or the controls. Reduced FC values in the posterior cingulate cortex (PCC) were also observed in the PD patients relative to the controls. Moreover, abnormal FC values of the right DN were observed throughout the frontal, parietal, and cingulate cortices by ANCOVA analysis (**Table 3** and **Figure 2**). Decreased FC values in the left precentral gyrus (M1) and right DLPFC were shown in MSA-P patients compared with the controls. PD patients produced attenuated connectivity in the PCC, medial PFC and bilateral DLPFC compared to the controls, as well as decreased FC values of the PCC compared to MSA-P.

**Table 3:** Regions showing significant differences in DN FC among MSA-P, PD, and control group.

		MNI coordinates			
			
Brain region	Cluster size	*X*	*Y*	*Z*	*T*-value
**Left dentate nucleus**					
MSA-P < Controls					
Left dorsolateral prefrontal cortex	44	-33	48	3	-5.14
PD < Controls					
Left dorsolateral prefrontal cortex	70	-33	49	9	-4.57
Posterior cingulate cortex	33	-3	-32	38	-3.94
Left inferior parietal lobe	39	-34	-62	46	-4.59
Right inferior parietal lobe	34	39	-66	45	-3.88
PD < MSA-P					
Left inferior parietal lobe	38	-29	-68	45	-4.61
Right inferior parietal lobe	30	37	-70	45	-3.37
**Right dentate nucleus**					
MSA-P < Controls					
Left precentral gyrus	20	-51	9	39	-4.08
Right dorsolateral prefrontal cortex	26	48	30	24	-3.95
PD < Controls					
Medial prefrontal cortex	27	3	42	39	-4.44
Left dorsolateral prefrontal cortex	28	-42	27	42	-3.89
Right dorsolateral prefrontal cortex	41	48	27	27	-4.04
Posterior cingulate cortex	180	-3	-36	33	-5.63
PD < MSA-P					
Posterior cingulate cortex	30	-9	-39	36	-3.82


*MNI, Montreal Neurological Institute; Significance set at *p* < 0.01 corrected by AlphaSim; Cluster size is in mm^*3*^; Results are given in MNI space.*

### Causal Connectivity

**FIGURE 3:**
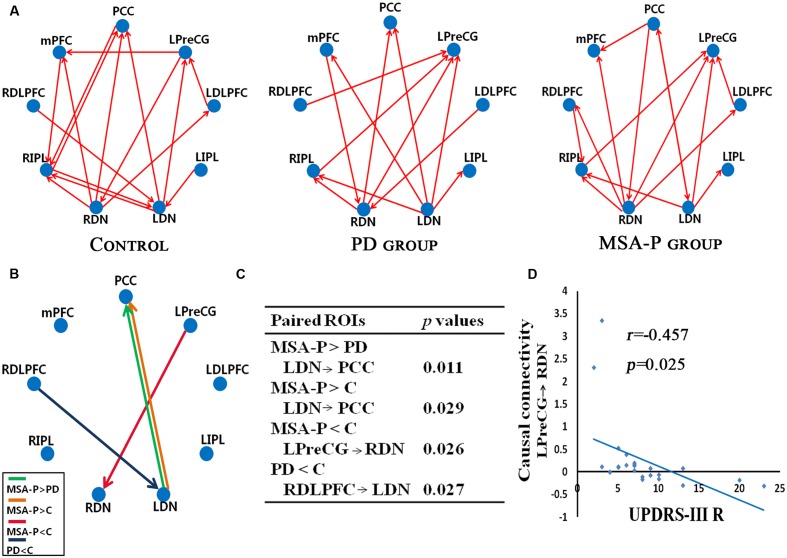
Granger causality (GC) analysis of alterations within the cerebello-cerebral circuits among the MSA-P, PD, and control groups. **(A)** Significant GC patterns within the controls, PD group and MSA-P group (*p* < 0.05). The red arrow indicates the direction of informational influence between paired regions; **(B)** significant differences in GC values between the groups: MSA-P versus controls, PD versus controls and MSA-P versus PD; **(C)** the *p*-value of each paired region is presented; **(D)** significant correlation between causal connectivity and UPDRS-III R scores in MSA-P group; PCC, posterior cingulate cortex; mPFC, medial prefrontal cortex; LDLPFC, left dorsolateral prefrontal cortex; RDLPFC, right dorsolateral prefrontal cortex; LPreCG, left precentral gyrus; LIPL, left inferior parietal lobe; RIPL, right inferior parietal lobe; LDN, left dentate nucleus; RDN, right dentate nucleus.

We further attested that the DN, as core regions of the neural circuitry, is causally influenced in the cerebello-cortical circuits. As shown in **Figure 3**, MSA-P and PD patients presented with different patterns of the connectivity strength of causal flow between the previously defined paired regions compared to controls. MSA-P patients showed significantly enhanced causal information from the left DN to the PCC compared to PD or controls, as well as lower information flow from the left M1 to the right DN relative to the controls. In the PD group, the right DLPFC exhibited obvious causal interaction disruption with the left DN compared to the controls, as shown in **Figure 3B**.

### Correlation Analysis with Clinical Behavior Scores

As shown in **Figure 3D**, the connectivity of the causal path from the left M1 to the right DN was significantly decreased in the MSA-P group, which was negatively correlated with UPDRS-III R scores.

## Discussion

Recently, many efforts have been made to detect early differences between MSA-P and PD patients (Wang et al., 2012; Ji et al., 2015). The novelty of this study lies in the fact that we have used unidirectional and directional connectivity methods to explore the unique association between altered cerebello-cortical connectivity and motor and non-motor defects in both MSA-P and PD patients. Our research yielded several major findings. First, MSA-P an PD patients exhibited both functional and causal connectivity differences within cerebello-cortical networks compared with controls. Second, the comparison of MSA-P patients with PD patients by BOLD signal mainly revealed differences in the DN-PCC connectivity. Third, in the MSA-P group, a significant correlation was found between the UPDRS-III R scores and the causal connectivity from the left M1 to right DN.

Relative to the controls, MSA-P patients displayed disrupted functional and causal coupling between the left M1 and right DN. The classical view of M1, which was based principally on direct cortical stimulation and attributed to select the muscles and force for executing an intended movement. A recent diffusion tensor imaging study has reported stronger connections between the cerebellum and the precentral gyrus as well as the superior frontal gyrus (Doron et al., 2010), which indicated that the cerebellum involved in the processing of motor, oculomotor, and spatial working memory by cerebello-cortical circuits (du Boisgueheneuc et al., 2006). Rs-fMRI in MSA has shown reduced regional homogeneity of the spontaneous BOLD fluctuations in left M1 compared with controls (You et al., 2011). Wang H. et al. (2016) also found that the induced motor improvement in MSA-P patients by rTMS over left M1 may be associated with increased activation in the cerebellum. The cerebellum receives input from the M1 and then projects to thalamus, M1 (Kelly and Strick, 2003). In this study, the causal influence from M1 to the cerebellum in MSA-P patients was attenuated. This result could imply that the cerebellum exerts its influence on cortical inhibitory activity (Picazio and Koch, 2015). It may be one explanation for the underlying pathophysiology of motor deficits. Noticeably, the decreased causal connectivity from left M1 to right DN is significantly correlated with the contralateral motor symptom scores for the MSA-P group, which emphasized that the motor impairments in MSA-P patients could be influenced by cerebello-cortical loop degeneration in addition to striatal pathology.

In this study, the cerebellar functional connectivity was disrupted in several default mode network (DMN) regions (the PCC, medial PFC, and inferior parietal lobe) in PD patients compared with MSA-P patients or controls. In the baseline state, the PCC appears a high metabolic rate (Raichle et al., 2001) and plays a crucial role in modulating the balance between internal and external information for maintaining normal brain functions (Leech and Sharp, 2014). Lin et al. (2016) found that the PCC is a core node of the DMN and it is closely related with cognitive task performance, by aggregating information to allow functional cooperation within the DMN. A previous study reported that cognitively unimpaired patients with PD tend to show attenuated DMN activity compared to controls (Tessitore et al., 2012). The study associated DMN deficits with cognitive decline, because cognitive function was correlated with DMN connectivity. It suggested that there is an early functional disruption of the DMN in PD prior to clinical evidence of cognitive impairment. The DMN sub-regions play important roles in remembering, self-reflection, mental imagery, and stream-of-consciousness processing (Greicius et al., 2004; Buckner and Carroll, 2007). The consolidation and maintenance of brain function might be facilitated through the DMN plasticity. However, the mechanisms underlying modulations of the DMN are not yet clear. We hypothesized that the DN dysfunction contributed to abnormal recruitment of the PCC node of the DMN. The DN, which represents the capital cerebellar output channel, is considered to be implicated with salience and sensorimotor networks (Habas et al., 2009). The DMN deactivates during externally goal-directed activity (Gusnard et al., 2001) and is causally influenced by the salience network (Chiong et al., 2013). In a recent report, a whole-brain functional connectivity analysis method was used to uncover connectivity changes following rTMS intervention over M1 for MSA-P patients. This result indicated that the rTMS-related functional links were mainly connected to the cerebellar and limbic networks from the DMN (Chou et al., 2015). It seems plausible that DMN plasticity might be sensitive to the cerebellar network effects. Only one study has described reduced DMN activity in MSA-P (with a average disease duration of 3.9 years) (Franciotti et al., 2015). In this study, MSA-P patients showed significant strengthened causal connectivity from the left DN to the PCC compared with both PD patients and controls. The DN exerted a causal influence on the PCC, perhaps as a compensatory mechanism for DMN functions in the early stages. In previous study, the DMN activity was enhanced in dementia with lewy bodies (DLB), the explanatory hypothesis was that the preserved DMN activity could depend on compensatory mechanisms attempting to maintain DMN functions, in the face of developing pathology (Kenny et al., 2012). The consolidated DMN connectivity was similar to the enhancement observed in DLB, as we found no cellular loss, and this finding supports that functional modulatory mechanisms are relevant rather than structural differences.

The inferior parietal lobe within the executive network is involved with sustained attention and working memory information for action preparation (Seeley et al., 2007). In patients with PD, the functional parieto-motor impairment could be related to bradykinesia (Palomar et al., 2013). The cerebellar damage may impair the competence to convert a programmed motion sequence into action before the launch of movement (Bhanpuri et al., 2014; Brunamonti et al., 2014). The interruption of the dynamic equilibrium between the cerebellar and executive network may weaken the ability of the motor system to prepare for future task execution. Evidence from previous studies has demonstrated the existed functional connectivity between the cerebellum and parietal cortex (Macher et al., 2014). The decline in functional connectivity between the DN and the inferior parietal lobe may contribute to the impaired function of PD patients in linking simple movements together into complicated and sequential movements (Liu et al., 2013). However, such correlations between clinical observations and neural connectivity patterns need further explorations.

Both MSA-P and PD patients showed decreased connectivity between the DN and the DLPFC compared with the controls. Reduced causal information flows from the DLPFC to the DN were also found in the PD group compared to the controls. The DLPFC, a crucial node in the cognition control network (Wang Y.L. et al., 2016), is involved in attention, working memory and executive control (Pope et al., 2015). MSA patients presented with a distinctive pattern of cognitive deficits in frontal executive dysfunction (Siri et al., 2013). A PET study suggested that MSA patients with memory and frontal executive dysfunction tended to show hypometabolism in the anterior cerebellum and frontal cortex in the early stage of the disease (Lyoo et al., 2008). In addition, the hypoactivity of the DLPFC in PD patients with depression has also been identified in previous studies (Zhu et al., 2016). The impaired striatal cells in parkinsonism could lead to secondary frontal lobe dysfunction, including disruption of the cognitive loop linking the striatum with the DLPFC (Jokinen et al., 2013). Recently, the possibility of cerebellar influence on cognitive function in parkinsonism has also been raised (Hirata et al., 2006; Wu and Hallett, 2013; Kim et al., 2015). Abundant structural and functional investigations in both human and non-human primates revealed that the cerebellum is involved in higher-order cognitive and emotional processes by sending fibers from the DN to PFC via the thalamus (Allen et al., 2005; Ramnani, 2006). The decreased DLPFC connectivity may be significantly associated with executive control and emotional processes in both MSA-P and PD patients, although no significant correlations were obtained in this study. These results illustrated that the connectivity of the cerebellum with the motor and non-motor cortical domains is significantly involved in the PD and MSA-P disease process.

This analysis demonstrated the cerebellum to be a causal flow hub of the cerebello-cortical network, with the high number of causal flow connections. A possible correspondence of tractography between cortices with similar functional roles, as reported here, suggests that the cerebellum contributes to parallel associative cerebello-cortical networks involved in various aspects of motor and cognition. The converging results strongly indicated that the causal topology of the cerebello-cortical circuits may be disrupted in both MSA-P and PD patients, adding an additional hint for comprehending the neurobiology underlying patients with MSA-P and PD.

## Conclusion

This rs-fMRI study provides evidence that the dysfunction reported within the cerebello-cortical networks, typically related to motor and cognitive defects in MSA-P and PD. It may be associated with impaired interactions between the cerebellum and key cerebral cortical regions. In conclusion, our findings indicate a crucial role for the cerebello-cortical network in both MSA-P and PD patients in addition to STC network and revealed that different patterns of cerebello-cortical loop degeneration are involved in the development of the diseases. Furthermore, the alterations of the functional link within the cerebello-cortical circuits, especially the DN-PCC connectivity, may facilitate early differential diagnosis and the monitoring of disease progression.

## Author Contributions

JS designed the study and revised it critically for important intellectual content. QY performed the research and drafted the manuscript, DZ and FL helped in data analyses, XL, CX, and QH help in clinical data collection, analyses and made patients follow-ups.

## Conflict of Interest Statement

The authors declare that the research was conducted in the absence of any commercial or financial relationships that could be construed as a potential conflict of interest.

## Footnotes

1 http://www.ansir.wfubmc.edu

2 http://www.rest.restfmri.net

3 http://www.fil.ion.ucl.ac.uk/spm/software/spm8
